# GPT Fusion: Reasoning-Based Integration of Specialized Convolutional Neural Networks for Melanoma Diagnosis

**DOI:** 10.21203/rs.3.rs-10500601/v2

**Published:** 2026-08-17

**Authors:** Katie L. Frederickson, Dong Li, Oren D. Edrich, Ashish C. Bhatia, Samuel E. Adunyah, Qingguo Wang

**Affiliations:** 1 Department of Biochemistry, Cancer Biology, Neurosciences and Pharmacology, School of Medicine, Meharry Medical College, Nashville, TN 37208, USA; 2 Harris School of Public Policy, University of Chicago, Chicago, IL 60637, USA; 3 Northwestern University Feinberg School of Medicine, Chicago, IL 60637, USA; 4 Rush University School of Medicine, Chicago, IL 60612, USA

**Keywords:** melanoma diagnosis, dermoscopy, convolutional neural network, large language model, ChatGPT

## Abstract

**Background:**

Malignant melanoma (MM) is the most aggressive form of metastatic skin cancer, for which early detection is critical to reducing morbidity and mortality while improving patient outcomes. Recent advances in multimodal large language models (LLMs) have demonstrated considerable potential for melanoma detection and clinical decision support. However, their diagnostic accuracy remains inferior to that of specialized convolutional neural networks (CNNs). Whether LLMs can effectively integrate predictions from specialized CNN models to improve melanoma diagnosis has not been systematically investigated.

**Methods:**

We developed a hybrid diagnostic approach, termed GPT Fusion, in which GPT-5.5 serves as a reasoning-based fusion framework rather than an image classifier. Instead of directly analyzing lesion images, GPT-5.5 received structured outputs from two independently developed CNN models: a multimodal ResNet-50 model trained on the MILK10K dataset for multiclass skin lesion classification and the first-place 90-model SIIM-ISIC ensemble optimized for melanoma detection. GPT-5.5 integrated these complementary predictions to generate the final diagnosis. The framework was evaluated using the publicly available Derm7pt dataset. Performance was assessed for melanoma prediction (melanoma vs. non-melanoma), malignancy prediction (malignant vs. benign lesions), primary diagnosis, and top-3 differential diagnosis.

**Results:**

GPT Fusion substantially outperformed image-based GPT-5.5 across all evaluation tasks. For melanoma prediction, accuracy improved from 63.4% to 86.9%, while ROC AUC increased from 0.750 to 0.923 and PR AUC from 0.547 to 0.853. For malignancy prediction, GPT Fusion not only exceeded the performance of image-based GPT-5.5 but also surpassed the specialized SIIM-ISIC ensemble in sensitivity (83.9%), F1 score (73.6%), ROC AUC (0.894), and PR AUC (0.823), while maintaining comparable overall accuracy. GPT Fusion further improved primary diagnosis accuracy and achieved the highest top-3 differential diagnosis accuracy (89.0%).

**Conclusions:**

These findings demonstrate that LLMs can provide greater clinical value as reasoning-based integrators of specialized AI systems than as standalone image classifiers. By combining the complementary strengths of specialized CNN models, GPT-5.5 substantially improved diagnostic performance while providing interpretable reasoning. This hybrid strategy offers a promising direction for developing more accurate, transparent, and clinically useful AI systems for melanoma diagnosis.

## 1. INTRODUCTION

Malignant melanoma (MM) is a high-risk neoplasm arising from melanocytes, the pigment-producing cells of the skin. It is the most aggressive form of skin cancer and has a high propensity for metastasis to regional and distant organs. The American Cancer Society (ACS) estimates approximately 112,000 new MM cases will be diagnosed in 2026, including approximately 65,400 cases in men and 46,600 in women [1]. An estimated 8,510 deaths are expected in the same year.

Early identification of melanoma is strongly associated with improved patient survival. However, accurate diagnosis remains challenging because early melanoma lesions often resemble benign pigmented lesions such as nevi or seborrheic keratoses. Diagnostic difficulty is further compounded by variations in skin pigmentation and lesion morphology across populations. To address these challenges, considerable research has focused on applying artificial intelligence (AI), in particular machine learning (ML) and deep learning (DL), to melanoma detection. Among DL approaches, convolutional neural networks (CNNs), deep learning models that automatically learn hierarchical visual features from images through successive convolutional layers, have become the dominant framework for image classification and lesion recognition. CNN-based architectures, such as Inception-v4, ResNet, DenseNet, and EfficientNet, have achieved dermatologist-level performance in landmark studies [2–5]. These advances have also translated into clinical practice, with AI-assisted tools such as FotoFinder’s Moleanalyzer-Pro and DermaSensor’s spectroscopy-based device supporting dermoscopic assessment [6, 7]. Despite their impressive diagnostic accuracy, however, these specialized CNN models function primarily as black-box classifiers, offering limited interpretability, clinical reasoning, and interactive decision support. This limitation extends beyond technical considerations: clinicians are less likely to trust diagnostic systems whose decision-making processes cannot be readily understood, and such lack of transparency has been widely recognized as a major barrier to the broader adoption of AI in clinical practice [8]. These limitations have motivated growing interest in AI systems that not only classify lesions accurately but also provide transparent reasoning, contextual interpretation, and interactive clinical decision support.

Timely melanoma detection is further complicated by limited access to dermatologic care in distant, rural areas. In the United States, dermatology residency programs offer only 574 positions annually, contributing to workforce constraints. A recent study reported a mean wait time of 50 days for a new patient dermatology appointment [9]. These barriers to timely dermatologic evaluation may contribute to delayed detection, disease progression, and increased melanoma-related morbidity and mortality. Therefore, AI-based diagnostic systems that expand access to melanoma screening and support of timely clinical diagnosis are urgently required.

Recent advances in large language models (LLMs) have introduced a new paradigm for medical AI. Unlike conventional CNNs, modern multimodal LLMs can integrate real-time visual, auditory, and textual information to generate diagnoses and natural-language explanations. Beginning with GPT-4 Vision (GPT-4V), LLMs have incorporated the ability to analyze user-uploaded medical images [10–12]. The recently released GPT-5 further improved diagnostic reasoning, robustness, and resistance to hallucinations across heterogeneous clinical inputs [10, 13, 14]. These developments position LLMs as promising tools for interactive clinical decision support that extend beyond image classification alone [15].

Accordingly, numerous studies have investigated the use of LLMs for melanoma detection. Shifai et al. evaluated GPT-4V using images from the International Skin Imaging Collaboration (ISIC) Archive [16]. Similarly, Liu et al. compared GPT-4V with Claude 3 Opus on the ISIC dataset [17]. Using the Human Against Machine with 10,000 training images (HAM10K) dataset, Sattler et al. evaluated GPT-4 Turbo (GPT-4T) and ChatGPT-4 Omni (GPT-4o), reporting variable diagnostic sensitivity, specificity, and accuracy [18]. Boostani et al. compared ChatGPT-4o and Gemini 2.0 Flash and found that their performance differed across clinical and dermoscopic images, and that combining the two models improved detection sensitivity [19]. More recently, Wang et al. evaluated GPT-5 comprehensively across the ISIC, HAM10K, and MILK10K datasets, demonstrating moderate performance in primary diagnosis and malignancy classification but substantially higher top-3 differential diagnosis accuracy [14, 20].

Collectively, these findings suggest that although current multimodal LLMs demonstrate considerable diagnostic potential, they remain insufficiently reliable as standalone melanoma classifiers. Conversely, specialized CNN models generally achieve higher diagnostic accuracy but lack the reasoning, contextual understanding, and interactive communication capabilities of LLMs. These complementary strengths motivate integrating the two approaches to develop a unified diagnostic framework, in which CNNs provide accurate image-based predictions, while LLMs synthesize CNN outputs, reconcile potentially conflicting predictions, incorporate relevant clinical information, and generate clinically meaningful diagnostic assessments through natural-language interaction with users.

In this study, we assessed a hybrid diagnostic strategy, termed GPT Fusion, in which GPT-5.5 functions as a reasoning-based meta-classifier that integrates the outputs of two specialized CNN systems: (i) a ResNet-50 model trained on the MILK10K dataset for broad skin lesion classification using paired clinical and dermoscopic images [21], and (ii) the first-place ensemble from the SIIM-ISIC Melanoma Classification Challenge, comprising multiple CNN models optimized specifically for binary melanoma detection [22]. Rather than classifying lesion images directly, GPT-5.5 in the framework serves as a reasoning-based diagnostic integrator by synthesizing the structured outputs of these specialized models to generate a consensus diagnosis and malignancy assessment. To our knowledge, this study represents the first systematic evaluation of an LLM that integrates state-of-the-art CNNs for melanoma diagnosis.

## 2. MATERIALS AND METHODS

**Figure 1.**
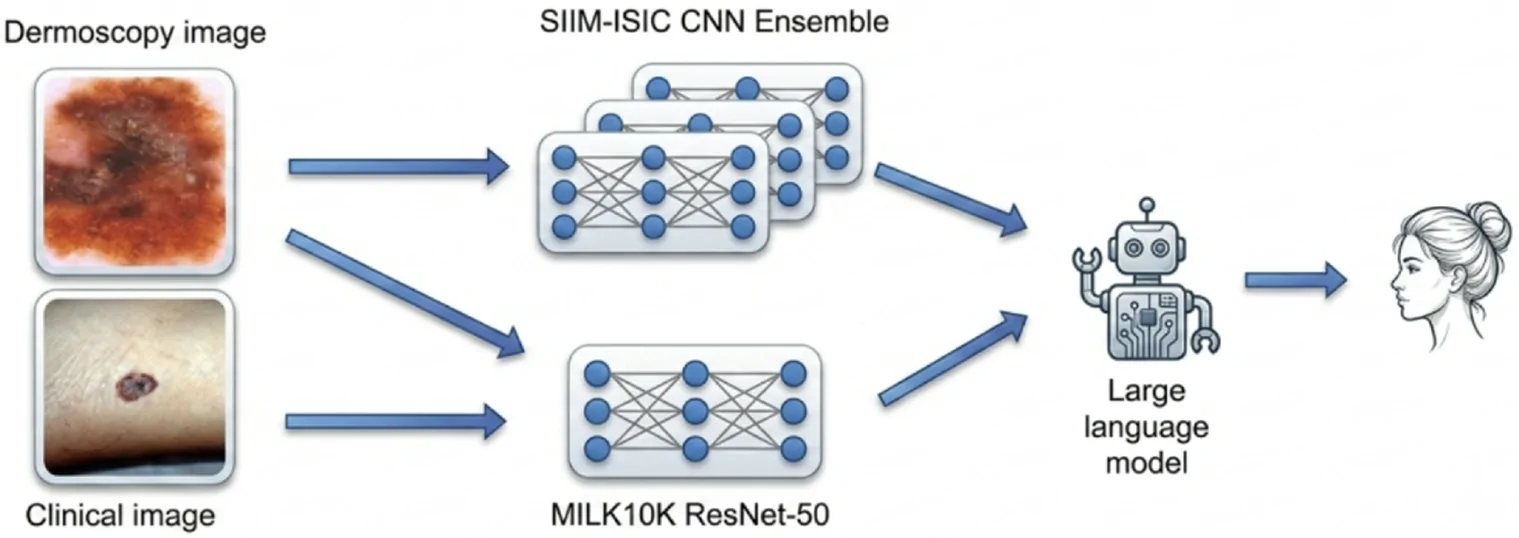
Overview of the proposed GPT Fusion framework. Dermoscopic images are analyzed by the SIIM-ISIC 90-model CNN ensemble, while paired dermoscopic and clinical images are analyzed by the ResNet-50 model trained on MILK10K. Rather than interpreting images directly, the LLM GPT-5.5 integrates the structured prediction outputs from both CNN models to generate a consensus diagnosis, differential diagnosis, melanoma probability, malignancy probability, and diagnostic rationale.

This study investigated whether an LLM can improve melanoma diagnosis by integrating the complementary strengths of specialized CNN models. Figure 1 provides an overview of the proposed GPT Fusion framework and the study workflow. Specifically, we compared four diagnostic paradigms: image-based GPT-5.5, a multimodal ResNet-50 model, the SIIM-ISIC CNN ensemble, and the proposed GPT Fusion framework, using the publicly available Derm7pt dataset [23]. Dermoscopy, the most widely adopted and clinically validated imaging modality for melanoma detection [24], served as the primary imaging modality throughout this study.

### 2.1 Derm7pt Dataset

All evaluations were performed using the Derm7pt dataset [23], which contains 1011 pairs of clinical close-up and dermoscopic images with expert-confirmed histopathological or clinical reference diagnoses and comprehensive clinical metadata. The dataset also includes annotations based on the seven-point checklist, facilitating the development and evaluation of computer-aided diagnostic systems. Derm7pt was selected as an independent external benchmark because neither the widely used ISIC Archive nor the MILK10K datasets were suitable for evaluation, as both had been used to train the CNN models assessed in this study.

The reference diagnosis provided in the metadata served as the ground truth for all performance evaluations. Because the MILK10K ResNet-50 model was trained using a different diagnostic taxonomy and the SIIM-ISIC CNN ensemble was designed exclusively for binary melanoma classification, diagnostic labels were harmonized before comparative analyses, and all diagnoses were mapped to the standardized diagnostic categories used in Derm7pt.

### 2.2 Image-Based CNN Models

#### ResNet-50.

Because the MILK10K investigators did not publicly release pretrained model weights, we reproduced their ResNet-50 model using the authors’ publicly available code and dataset without modification [21]. The model was trained on MILK10K following the original training protocol, and its performance was verified on the MILK10K test set (Supplementary eFigure 1 and eTable 1). The validated model was then applied directly to the Derm7pt dataset without further fine-tuning or parameter optimization. The multimodal ResNet-50 model accepts paired clinical close-up and dermoscopic images and predicts 48 fine-grained skin lesion diagnoses together with calibrated class probabilities. To facilitate clinical interpretation, the MILK10K investigators grouped these 48 diagnoses into 11 broader diagnostic categories, with each detailed diagnosis assigned to a single category. For comparative evaluation in this study, the ResNet-50 predictions were further mapped to the harmonized Derm7pt diagnostic categories.

#### SIIM-ISIC CNN Ensemble.

The publicly released model checkpoints from the first-place solution of the SIIM-ISIC Melanoma Classification Challenge were downloaded directly from the Kaggle repository and evaluated without retraining or modification (Supplementary eFigure 2 and eTable 2) [22]. The winning solution employed 18 distinct model configurations, primarily based on EfficientNet and ResNeXt architectures operating at multiple image resolutions. Each configuration was trained using 5-fold cross-validation, resulting in an ensemble of 90 trained model checkpoints (hereafter referred to as the SIIM-90 Ensemble). Each checkpoint generated a continuous probability of melanoma for each lesion. Predictions from the checkpoints were combined using two ensemble strategies: (i) a probability ensemble, in which melanoma probabilities were averaged across all checkpoints, and (ii) the official SIIM rank ensemble, which aggregates normalized prediction rankings across model configurations.

### 2.3 Reasoning-Based Diagnostic Fusion

To integrate the complementary strengths of CNNs and LLMs, we have LLMs (GPT-5.5, a leading LLM, was used in this study) serve as a reasoning-based meta-classifier rather than an image classifier. Unlike the image-based assessment, GPT-5.5 was not provided with either the clinical close-up or dermoscopic images. Instead, it received only the structured textual and numerical outputs generated by the ResNet-50 and SIIM-90 ensemble models, including the primary diagnosis, top-3 differential diagnoses, class probabilities, melanoma probability, malignancy probability, and threshold-adjusted binary predictions.

The standardized prompt explicitly informed GPT-5.5 of the complementary roles of the two AI models. Specifically, the SIIM-90 ensemble was described as optimized for melanoma detection and therefore should receive greater weight when estimating melanoma probability, whereas the ResNet-50 model was described as providing broader multiclass diagnostic information and should primarily contribute to differential diagnosis and the assessment of non-melanoma malignancies. GPT-5.5 was instructed to integrate these complementary predictions to generate a consensus diagnosis.

### 2.4 Diagnostic Tasks

Two binary classification tasks were evaluated. Melanoma prediction assessed the ability to distinguish melanoma from all other skin lesions, including both benign lesions and non-melanoma malignancies. In contrast, malignancy prediction assessed the ability to distinguish all malignant skin lesions (e.g., melanoma, basal cell carcinoma, squamous cell carcinoma, and other malignant tumors) from benign lesions. Thus, melanoma prediction assesses performance on a disease-specific task, whereas malignancy prediction reflects the broader clinical objective of identifying lesions that require prompt diagnostic evaluation and treatment.

In addition to the two binary classification tasks, diagnostic performance was evaluated using top-1 primary diagnosis and top-3 differential diagnosis after mapping lesion diagnoses to standardized diagnostic categories. Top-1 primary diagnosis accuracy represents the percentage of lesions for which the model’s first (highest-confidence) diagnosis is correct. Top-3 differential diagnosis accuracy represents the percentage of lesions for which the correct diagnosis is included among the model’s three highest-ranked candidate diagnoses. Whereas top-1 accuracy evaluates the ability to identify the single best diagnosis, top-3 accuracy reflects the ability to generate an appropriate differential diagnosis, a process that closely resembles clinical practice in which physicians consider several plausible diagnoses before confirming the final diagnosis.

### 2.5 Performance Evaluation

For these diagnostic tasks, performance was evaluated using accuracy, sensitivity, specificity, precision, F1 score, receiver operating characteristic area under the curve (ROC AUC), and precision-recall area under the curve (PR AUC). For the ResNet-50 and SIIM-90 models, optimal decision thresholds were determined by maximizing the Youden index (*J*), *J* = *Sensitivity* + *Specificity* - *1*, which identifies the threshold that jointly maximizes sensitivity and specificity. The optimized thresholds were subsequently fixed and applied throughout all LLM fusion experiments.

### 2.6 Implementation

The complete evaluation pipeline was implemented in Python. Model outputs were parsed, normalized, and benchmarked against the Derm7pt reference standards. Continuous prediction scores were utilized to generate ROC and precision-recall curves, while binary metrics were evaluated using either the optimized operating thresholds or model-generated binary decisions, as appropriate. The source code developed for this study, including evaluation scripts for the ResNet-50 model, the SIIM-ISIC ensemble, and the LLM-based diagnostic fusion framework, is publicly available at https://github.com/qwangmsk/Melanoma-Detect.

## 3. RESULTS

### 3.1 Melanoma Prediction

**Figure 2.**
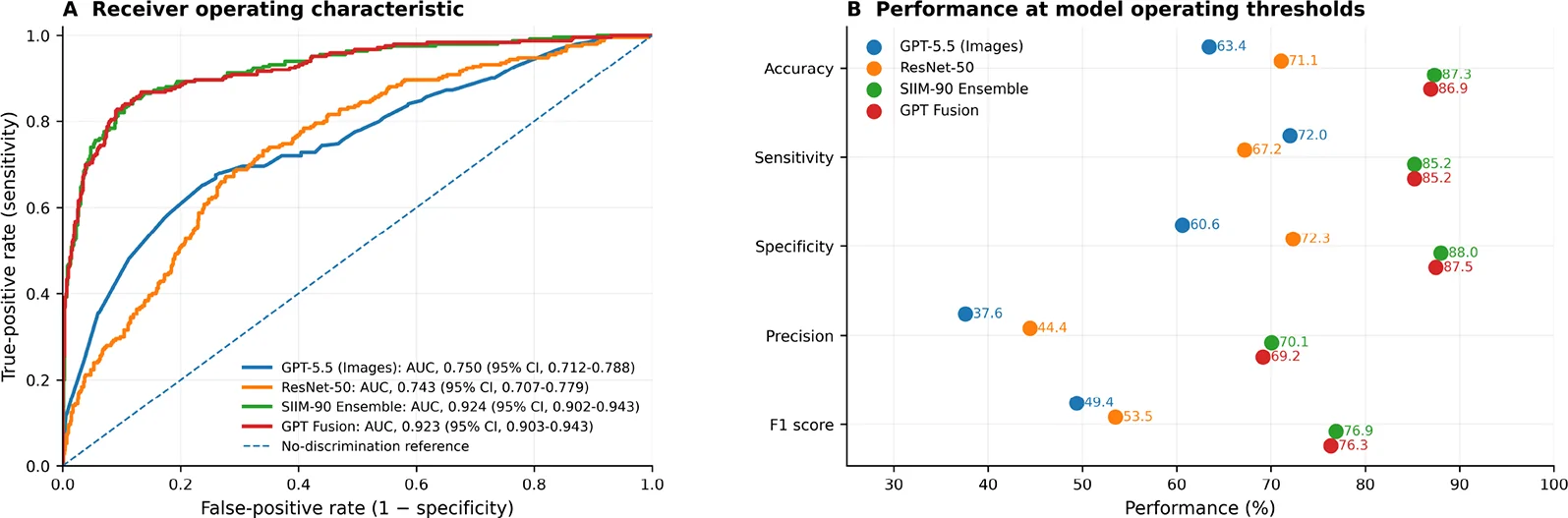
Comparative melanoma prediction performance of four AI approaches on the Derm7pt dataset. Analyses included the 1009 Derm7pt lesions for which predictions were available from all four approaches. **A**, Receiver operating characteristic (ROC) curves for GPT-5.5 (Images), ResNet-50, the SIIM-90 CNN ensemble, and GPT Fusion. Areas under the ROC curves (AUCs) and 95% confidence intervals were estimated using 2,000 bootstrap resamples. **B**, Comparison of threshold-dependent diagnostic performance, including accuracy, sensitivity, specificity, precision, and F1 score. Metrics were calculated using the prediction decisions generated by GPT-5.5 (Images) and GPT Fusion and the predefined operating thresholds for ResNet-50 and the SIIM-90 ensemble. Operating thresholds for the CNN models were established during model validation and are described in the Supplement.

GPT Fusion and the SIIM-90 ensemble substantially outperformed image-based GPT-5.5 and ResNet-50 for melanoma prediction (Table 1; Figure 2). GPT Fusion achieved the highest sensitivity and PR AUC, whereas the SIIM-90 ensemble showed slightly higher accuracy, specificity, precision, F1 score, and ROC AUC. Relative to image-based GPT-5.5, GPT Fusion improved every threshold-dependent performance metric while increasing ROC AUC from 0.750 to 0.923 and PR AUC from 0.547 to 0.853, demonstrating that reasoning-based integration of complementary CNN predictions markedly enhances melanoma discrimination.

### 3.2 Malignancy Prediction

**Figure 3.**
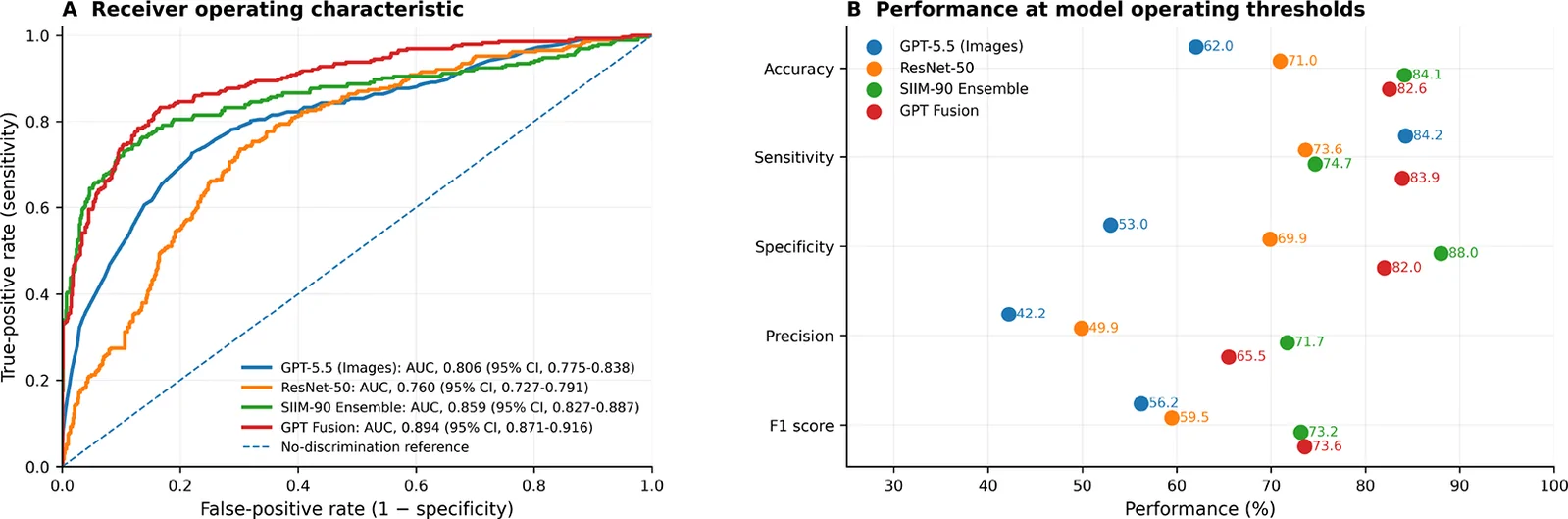
Comparative malignancy prediction performance of four AI approaches on the Derm7pt dataset. Analyses included the 1009 Derm7pt lesions for which predictions were available from all four approaches. **A**, Receiver operating characteristic curves for GPT-5.5 (Images), ResNet-50, the SIIM-90 ensemble, and GPT Fusion. Areas under the receiver operating characteristic curves and 95% confidence intervals were estimated using 2000 bootstrap resamples. **B**, Comparison of threshold-dependent diagnostic performance, including accuracy, sensitivity, specificity, precision, and F1 score. Metrics were calculated using the prediction decisions generated by GPT-5.5 (Images) and GPT Fusion and the predefined operating thresholds for ResNet-50 and the SIIM-90 ensemble. Operating thresholds for the CNN models were established during model validation and are described in the Supplement.

Whereas melanoma prediction focuses specifically on identifying melanoma, malignancy prediction evaluates the broader clinical task of distinguishing malignant from benign lesions. Accordingly, this task provides a more comprehensive assessment of the clinical utility of the proposed GPT Fusion framework. Performance for all four approaches is summarized in Table 2 and Figure 3.

GPT Fusion and the SIIM-90 ensemble substantially outperformed image-based GPT-5.5 and ResNet-50 for malignancy prediction (Table 2; Figure 3). GPT Fusion achieved the highest F1 score (73.6%), ROC AUC (0.894), and PR AUC (0.823), whereas the SIIM-90 ensemble achieved slightly higher accuracy (84.1%), specificity (88.0%), and precision (71.7%). Compared with image-based GPT-5.5, GPT Fusion improved accuracy by more than 20 percentage points while substantially increasing specificity, precision, F1 score, ROC AUC, and PR AUC, with comparable sensitivity.

### 3.3 Diagnostic category classification performance

**Figure 4.**
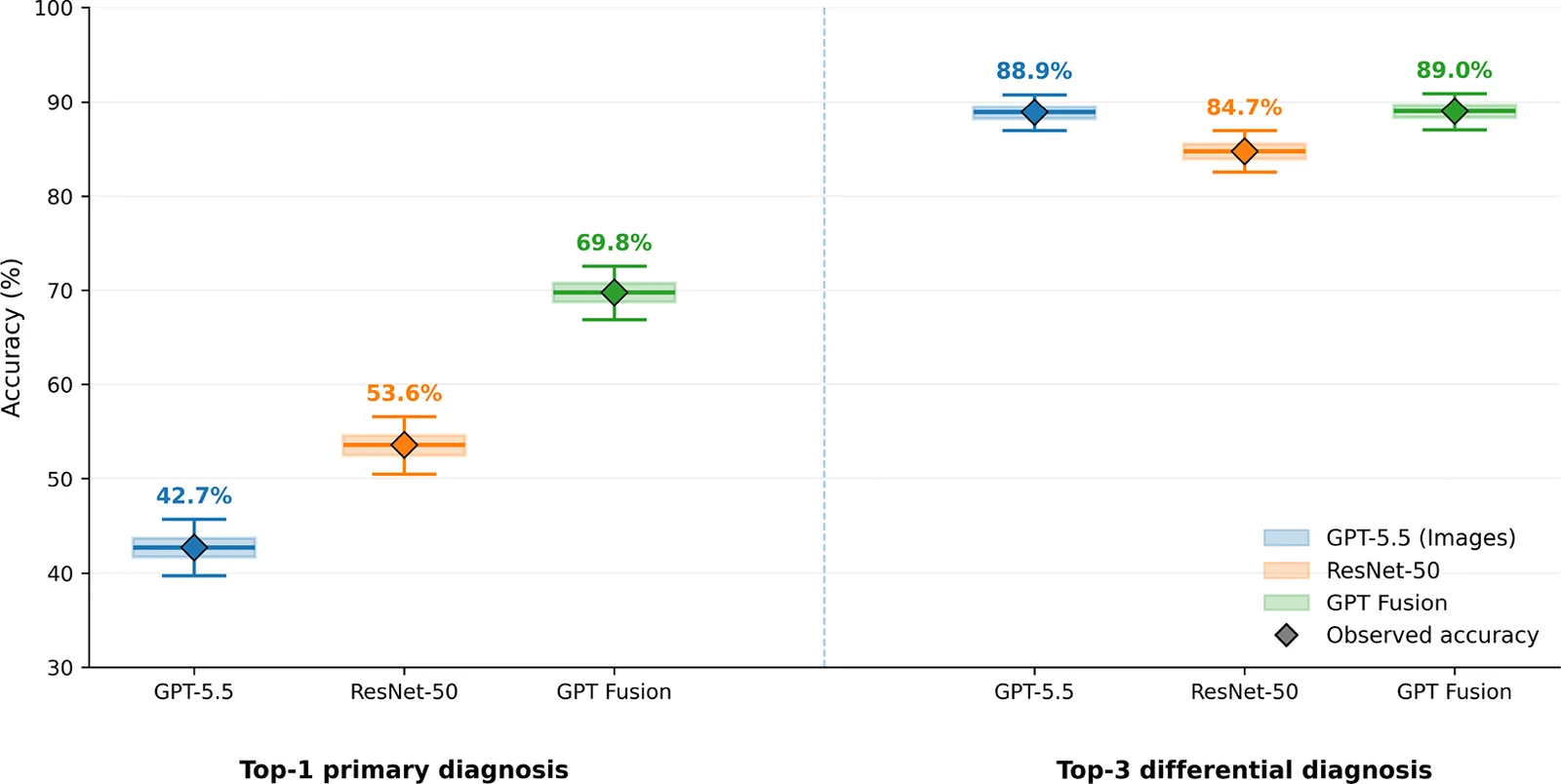
Comparison of diagnostic category classification performance across three AI approaches on the Derm7pt dataset. Diagnostic performance was evaluated for **Top-1 primary diagnosis** and **Top-3 differential diagnosis** using the 1,009 Derm7pt lesions with predictions available from all three approaches. Boxplots represent the distribution of classification accuracy estimated from 2,000 bootstrap resamples of the evaluation cohort. Center lines indicate medians; boxes, interquartile ranges; whiskers, minimum and maximum values; diamonds, observed accuracy calculated from the original dataset. Top-1 accuracy denotes correct primary diagnosis, whereas Top-3 accuracy denotes inclusion of the reference diagnosis among the three highest-ranked predictions.

GPT Fusion achieved the highest primary diagnosis accuracy (69.8%), substantially outperforming GPT-5.5 (42.7%) and ResNet-50 (53.6%) (Figure 4). Improvements in top-3 differential diagnosis were modest because GPT-5.5 already demonstrated strong differential diagnostic performance. The SIIM-90 ensemble was excluded because it produces only binary melanoma predictions and does not generate diagnostic categories.

## 4. DISCUSSION

AI has transformed melanoma diagnosis over the past decade. Specialized CNN models have achieved dermatologist-level performance on benchmark datasets and have been incorporated into clinical decision-support systems. More recently, multimodal LLMs have demonstrated the ability to interpret medical images, provide natural-language explanations, and support interactive clinical decision-making. These two classes of AI systems exhibit complementary strengths: CNNs excel at image pattern recognition but generally function as black-box classifiers, whereas LLMs provide reasoning capability, contextual understanding, and interactive communication but currently remain less accurate than specialized CNNs for melanoma diagnosis. This study investigated whether these complementary strengths could be combined through a reasoning-based diagnostic fusion strategy.

Our findings demonstrate that GPT Fusion consistently improved upon image-based GPT-5.5 across melanoma prediction, malignancy prediction, and diagnostic category classification while achieving performance comparable to, and for several metrics exceeding, the strongest specialized CNN benchmark. Rather than functioning as a conventional image classifier, GPT-5.5 served as a reasoning-based diagnostic integrator that synthesized structured outputs generated by specialized CNN models. These findings suggest that the greatest clinical value of current LLMs in dermatologic diagnosis may lie not in replacing specialized image-analysis models, but in orchestrating complementary AI systems and translating their outputs into clinically meaningful diagnostic decisions.

An important finding of this study is that the multimodal MILK10K ResNet-50 model remained a valuable contributor to GPT Fusion despite its relatively modest standalone performance on the external Derm7pt dataset. The lower performance of ResNet-50 likely reflects both the limited representational capacity of the ResNet-50 architecture and reduced domain generalization, as the model was trained exclusively on the standardized MILK10K dataset. Nevertheless, unlike the SIIM-90 ensemble, which was optimized specifically for binary melanoma detection, the MILK10K model provides multiclass predictions spanning a broad spectrum of benign and malignant skin lesions. By reasoning over these complementary outputs, GPT-5.5 preserved the excellent melanoma discrimination of the SIIM-90 ensemble while incorporating broader diagnostic information unavailable from the melanoma-specific model alone.

The complementary strengths of these CNN models were most evident in malignancy prediction. Whereas melanoma prediction focuses on identifying a single cancer subtype, malignancy prediction requires distinguishing multiple malignant lesion types from benign lesions. Consistent with this broader clinical objective, GPT Fusion achieved performance comparable to the SIIM-90 ensemble for overall accuracy while exceeding it in sensitivity, F1 score, ROC AUC, and PR AUC. These findings suggest that reasoning-based integration becomes increasingly advantageous as diagnostic tasks require synthesizing information from multiple specialized models rather than optimizing performance for a single disease.

The GPT Fusion framework differs fundamentally from conventional ensemble learning. Traditional ensemble methods combine model outputs mathematically through probability averaging, majority voting, or weighted combinations. Although these approaches often improve predictive performance, they cannot explicitly reconcile conflicting predictions, exploit complementary diagnostic information, or provide transparent explanations for their conclusions. In contrast, GPT Fusion performs semantic reasoning over structured outputs from specialized models, generating consensus diagnoses together with interpretable diagnostic rationales. By integrating complementary evidence through explicit reasoning rather than mathematical aggregation alone, GPT Fusion provides both competitive diagnostic performance and a transparent record of the information during decision-making.

Trust has been recognized as a persistent barrier to the clinical adoption of AI[8]. Sagona et al. argue that clinician trust depends less on assumptions about AI intent than on whether a system produces consistent, predictable, and reliable results [8]. GPT Fusion addresses this dimension of trust in two complementary ways. First, it demonstrated robust performance on an external dataset, substantially outperforming image-based GPT-5.5 across all evaluated tasks and surpassing the specialized SIIM-90 ensemble in sensitivity, F1 score, ROC AUC, and PR AUC for malignancy prediction. Second, GPT Fusion generates natural-language diagnostic rationales that document how evidence from complementary AI models contributes to the final decision. Importantly, we do not interpret these rationales as evidence that the underlying reasoning is inherently faithful or reliable, a stronger claim that remains the subject of ongoing investigation [25, 26]. Rather, consistent with the perspective of Sagona et al., the principal basis for clinician trust should remain demonstrated diagnostic performance, while interpretable rationales may facilitate clinician understanding, communication with patients, and retrospective audit without serving as independent evidence of reliability [8].

Although demonstrated here using melanoma diagnosis, GPT Fusion represents a general reasoning framework for integrating heterogeneous AI systems. The framework is independent of any specific CNN architecture or medical specialty and can readily incorporate additional diagnostic models, foundation models, or other domain-specific AI systems. Similar reasoning-based orchestration strategies may therefore prove valuable in pathology, radiology, ophthalmology, dentistry, and other biomedical domains where multiple complementary AI models are increasingly available.

Several limitations should be acknowledged. First, the proposed framework was evaluated using a single external dataset, and validation across larger, more diverse clinical cohorts will be necessary to establish generalizability. Second, only one LLM and two CNN models were investigated. Future studies should determine whether similar improvements can be achieved using alternative LLMs and additional specialized dermatologic AI systems. Third, we did not evaluate the quality or faithfulness of the diagnostic rationales generated by GPT Fusion. It remains unclear whether these explanations accurately reflect the underlying decision process or whether they improve clinician performance without inducing unwarranted confidence. Accordingly, we regard the rationales as records that may support retrospective audit rather than as independent grounds for clinical reliance. Future work should therefore evaluate explanation quality, prospective clinical performance, more sophisticated multi-agent reasoning strategies, and integration into routine dermatologist workflows.

Overall, these findings support a shift in how LLMs are deployed in medical AI. Rather than competing directly with highly optimized disease-specific image classifiers, LLMs may contribute most effectively as reasoning engines that integrate complementary evidence from multiple specialized AI systems. As increasingly powerful foundation models and domain-specific diagnostic algorithms become available, reasoning-based orchestration frameworks such as GPT Fusion may provide a practical path toward more accurate, interpretable, trustworthy, and clinically useful AI-assisted diagnosis.

## 5. CONCLUSIONS

This study assessed GPT Fusion, a hybrid melanoma diagnosis framework that uses GPT-5.5 as a reasoning-based meta-classifier to integrate predictions from specialized CNN models rather than directly interpreting lesion images. By combining the complementary strengths of a melanoma-specialized SIIM-90 ensemble (trained on the ISIC Archive) and a multimodal ResNet-50 model (trained on MILK10K), GPT Fusion substantially improved diagnostic performance over image-based GPT-5.5 and achieved performance comparable to or better than the specialized CNN models across several clinically relevant tasks. These findings suggest that the greatest clinical value of LLMs in dermatologic diagnosis may lie not in replacing specialized image-analysis models, but in orchestrating and interpreting their outputs through reasoning-based integration. More broadly, GPT Fusion illustrates a general paradigm in which an LLM functions as a reasoning-based orchestrator of heterogeneous AI systems. Although demonstrated here for melanoma diagnosis, the approach is readily extensible to other multimodal biomedical and scientific applications requiring the integration of specialized predictive models.

## Supplementary Material

Supplementary Files

This is a list of supplementary files associated with this preprint. Click to download.

• supplementaryfigures.pdf

## Figures and Tables

**Table 1.** Comparative performance of GPT Fusion and three AI approaches for binary melanoma prediction on the Derm7pt dataset.

AI approach	Accuracy	Sensitivity	Specificity	Precision	F1	ROC AUC	PR AUC

GPT-5.5 (Images)	63.4%	72.0%	60.6%	37.6%	49.4%	0.750	0.547
ResNet-50	71.1%	67.2%	72.3%	44.4%	53.5%	0.743	0.492
SIIM-90 Ensemble	**87.3%**	**85.2%**	**88.0%**	**70.1%**	**76.9%**	**0.924**	0.850
GPT Fusion	86.9%	**85.2%**	87.5%	69.2%	76.3%	0.923	**0.853**

Values represent performance on the common subset of Derm7pt lesions for which predictions were available from all four approaches. Accuracy, sensitivity, specificity, precision, and F1 score were calculated using each model’s operating threshold. ROC AUC and PR AUC were calculated from continuous melanoma probabilities.

**Table 2.** Comparative performance of GPT Fusion and three AI approaches for binary malignancy prediction on the Derm7pt dataset.

AI approach	Accuracy	Sensitivity	Specificity	Precision	F1	ROC AUC	PR AUC

GPT-5.5 (Images)	62.0%	**84.2%**	53.0%	42.2%	56.2%	0.806	0.666
ResNet-50	71.0%	73.6%	69.9%	49.9%	59.5%	0.760	0.540
SIIM-90 Ensemble	**84.1%**	74.7%	**88.0%**	**71.7%**	73.2%	0.859	0.810
GPT Fusion	82.6%	83.9%	82.0%	65.5%	**73.6%**	**0.894**	**0.823**

Values represent performance on the common subset of Derm7pt lesions for which predictions were available from all four approaches. Accuracy, sensitivity, specificity, precision, and F1 score were calculated using each model’s operating threshold. ROC AUC and PR AUC were calculated from continuous malignancy probabilities.

## Data Availability

Derm7pt data are open for research use, and do not include proprietary or patient-identifiable data. All Derm7pt dermoscopic and clinical images, along with metadata, analyzed in this study are publicly available and can be assessed at https://github.com/jeremykawahara/derm7pt. Additionally, the Python scripts developed for this study, including code for processing images, will be made publicly available at https://github.com/qwangmsk/Melanoma-Detect.
